# Metabolic Syndrome in Patients With Diabetes Mellitus

**DOI:** 10.7759/cureus.24469

**Published:** 2022-04-25

**Authors:** Mohammed Amine Essafi, Latifa bouabdellaoui, Hayat Aynaou, Houda Salhi, Hanan El Ouahabi

**Affiliations:** 1 Endocrinology, Diabetology, Metabolic Diseases and Nutrition, Hassan II University Hospital Center, Fez, MAR

**Keywords:** diabete type 2, diabete type 1, degenerative complications, metabolic syndrome, diabetes mellitus

## Abstract

Aim

Our study aims to assess the prevalence of metabolic syndrome (MS) in patients with diabetes mellitus, identify its determinants, and determine the correlation between MS and degenerative complications.

Method

A retrospective, descriptive and analytic study was conducted at the Endocrinology, Diabetology, and Nutrition department of the Hassan II University Hospital in Fez, over the period between January 2009 and January 2019. We included in our study all type 1 and type 2 diabetic patients. The presence of metabolic syndrome was defined according to the criteria of the International Diabetes Federation (IDF) and American Heart Association/National Heart, Lung and Blood Institute (AHA / NHLBI) Joint Scientific Statement (2009). The data were entered in Microsoft Excel (Microsoft Corporation. 2018). and analyzed using SPSS software (IBM Corp. Released 2015. IBM SPSS Statistics for Windows, Version 23.0. Armonk, NY: IBM Corp).

Result

A total of 1034 patients were included in this study; 78.7% were type 2 diabetics (T2D) and 21.3% were type 1 diabetics (T1D). The average age was 52,06 ± 17,33 years in T2D and 27,04 ± 9,66 years in T1D. 65,5% were females. The prevalence of metabolic syndrome was 78.4% for T2D, and 27.3% for T1D. The most common abnormality was high blood pressure in T2D (87.7%), and visceral obesity in T1D (68.3%). The most common triad was the association of increased waist circumference, hypertension, and diabetes (in 25,5% of T2D and 20% of T1D). By comparing the population of diabetics with metabolic syndrome (MS) and those without, we noticed a significant difference (p<0.05) concerning age, gender, all components of MS (high blood pressure, abdominal obesity, hyper triglyceridemia (TG), and hypo high-density lipoprotein cholesterol (HDL-C). Diabetic retinopathy and ischemic heart disease were the only chronic complications correlated with MS (p <0.05).

Conclusion

High prevalence of metabolic syndrome in diabetic patients. Its screening and the specific treatment of its various components are essential in order to reduce the complications which jeopardize the functional as well as the vital prognosis of these patients.

## Introduction

Diabetes mellitus is a metabolic disease, its chronic complications make all its gravity. According to the International Diabetes Federation, the global diabetes prevalence in 2021 is estimated to be 10.5% (536.6 million people), rising to 12.2% (783.2 million) in 2045 [1]. The association of other factors increases the vascular risk in patients with diabetes, among which is the metabolic syndrome (MS) that precedes or accompanies diabetes [2]. This syndrome constitutes an entity that groups in the same individual several metabolic, clinical, and biological abnormalities. It increases the risk of cardiovascular disease threefold [3].

Several definitions have been proposed, making it difficult to estimate its true prevalence [4]. In general, the MS corresponds to the combination of visceral obesity, lipid disturbances, carbohydrate disturbances, insulin resistance, and hypertension [5]. The prevalence of MS depends on its definition, the year of study, age, sex, and ethnicity of the population studied. It is increasing rapidly in both developed and developing countries. This has been attributed to changes in lifestyle, particularly with regard to new eating habits and sedentary lifestyles [6].

In Morocco, there is no large study that can provide a reliable estimate of the prevalence of MS in patients with diabetes, hence the interest of our study. In this study, we propose to estimate the prevalence of MS and its different parameters in diabetics, to determine the correlation between this syndrome and chronic complications.

## Materials and methods

Study and population

A retrospective, descriptive and analytic study was conducted at the Endocrinology, Diabetology, and Nutrition department of the Hassan II University Hospital Centre in Fez, over the period between January 2009 and January 2019. We included in our study all adult patients with a confirmed diagnosis of type 1 (T1D) or type 2 diabetes (T2D). We excluded those with secondary diabetes, pregnant women, and incomplete records.

Data collection

The data were collected from the medical records of patients with diabetes, who were hospitalized at the Endocrinology, Diabetology, and Nutrition Department of the Hassan II University Hospital Center in Fez, and were reported on an exploitation form, then integrated into a computer database. The socio-demographic variables were age, gender, socioeconomic level, and smoking status. Clinical and anthropometric variables were diabetes history, clinical examination that consisted of blood pressure, weight, height, the body mass index (BMI), and waist circumference measurements. Paraclinical variables were fasting blood glucose, total cholesterol, high-density lipoprotein cholesterol (HDL-C), low-density lipoprotein cholesterol (LDL-C), triglycerides, search for chronic complications, glycated hemoglobin (HbA1c), uric acid, creatinine, creatinine clearance, 24-hour albuminuria, ophthalmologic evaluation, trans-thoracic echocardiography, echo-Doppler of the lower limbs, echography of the supra-aortic trunks, and other explorations in cases that require.

The metabolic syndrome was defined with other metabolic abnormalities than diabetes, according to the criteria of the joint scientific statement of the International Diabetes Federation (IDF) criteria in agreement with the American Heart Association/National Heart, Lung and Blood Institute (AHA/NHLBI) in 2009 [7]:

1. Abdominal obesity with a waist circumference greater than or equal to 94 cm for men or 80 cm for women.

2. Systolic blood pressure greater than or equal to 130/85 mmHg or known hypertension.

3. A triglyceride level greater than or equal to 150 mg/dl or specific treatment for the lipid abnormality.

4. Low high-density cholesterol (HDL-C) lipoprotein with a level less than 40 mg/dl for males and 50 mg/dl for females or specific treatment of the lipid abnormality.

Statistical analysis

Frequencies were measured for qualitative variables. Means and standard deviations were used for quantitative variables. The classical parametric test (chi2 test) was used to test associations between categorical variables. In all the analyses, the level of significance was kept at a p-value lower than 0.05. Statistical analysis was performed using SPSS software (IBM Corp. Released 2015. IBM SPSS Statistics for Windows, Version 23.0. Armonk, NY: IBM Corp).

Ethics statement

Anonymity and confidentiality were respected for all participants.

## Results

Clinical and biological characteristics

A total of 1034 subjects were included in the study, the majority of participants were diagnosed with diabetes type 2 (n 814, 78.7%) versus a minority with diabetes type 1 (n 220, 21.3%). In patients with type 2 diabetes, the mean age was 52.06 ± 17.33 years with a predominance of the age group (50-59 years) (31.20%), the majority of which was female (67.2%, n 547). The average BMI was 27.13 ± 5.58 kg/m²; overweight was objectified in 49.9% and 31.4% had obesity. A large waist circumference (LWC) was found in 69.4% (n 565). Hypertension was present in 77.5% of patients (n 631). A low HDL-C level in 58.3% (n 475). 37.9% of our T2D had hypertriglyceridemia (n 309).

In patients with type 1 diabetes, the mean age was 27.04 ± 9.66 years with a predominance of the age group (30-40 years) (32.5%), of which the majority was female (59.5%, n 131). The mean BMI was 22.6 ± 4.88 kg/m² (overweight was objectified in 43.2% and 5.9% had obesity). A large waist circumference (LWC) was found in 28.6% (n 63). Hypertension was present in 20.5% of patients (n 45). 40% had a low HDL-C level (n 88). and 18.2% had hypertriglyceridemia (n 40).

Metabolic syndrome in our patients

Prevalence of metabolic syndrome was 67.5% (n 698), it was higher among patients with type 2 diabetes (78.4% versus 27.3% in T1D) and among females (39.5%). The clinical and biological characteristics of patients with MS (MS+) and without (MS-) are summarized in Table 1. The omnipresent abnormality in T2D was high blood pressure (hypertension), found in 87.7%, and in T1D was visceral obesity (large waist circumference) in 68.3%. By comparing the population of diabetics with metabolic syndrome (MS+) and that without (MS-), we noticed a significant difference (p<0.05) concerning age, gender, and all components of MS (high blood pressure, abdominal obesity, hyperTG, and hypo HDL) (Table 1).

**Table 1:** Clinical and biological characteristics of diabetics with and without MS LWC: large waist circumference. HBP: High blood pressure, HDL-C: high-density lipoprotein cholesterol, TG: triglyceridemia

	Type 2 diabetics			Type 1 diabetics		
	MS+	MS-	p- value	MS+	MS-	p- value
	78.4% (n 638)	21.6% (n 176)		27.3% (n 60)	72.7% (n 160)	
Age	51.09 ±17.6	54,06 ±16.4	0.01	31.5 ± 8.2	26.1 ± 10.8	0.01
Gender	F: 68.7%	F: 62%	0.01	F: 70%	F: 55.6%	0.001
LWC	83%	19.8%	0.0003	68.3%	13.8%	0.0002
HBP	87.7%	40.3%	0.001	51.7%	8.8%	0.0001
Hypo HDL-C	57%	22.7%	0.002	58.3%	33.1%	0.001
Hyper TG	47.3%	3.9%	0.0001	56.7 %	3.8 %	0.0001

Chronic complications

**Figure 1:**
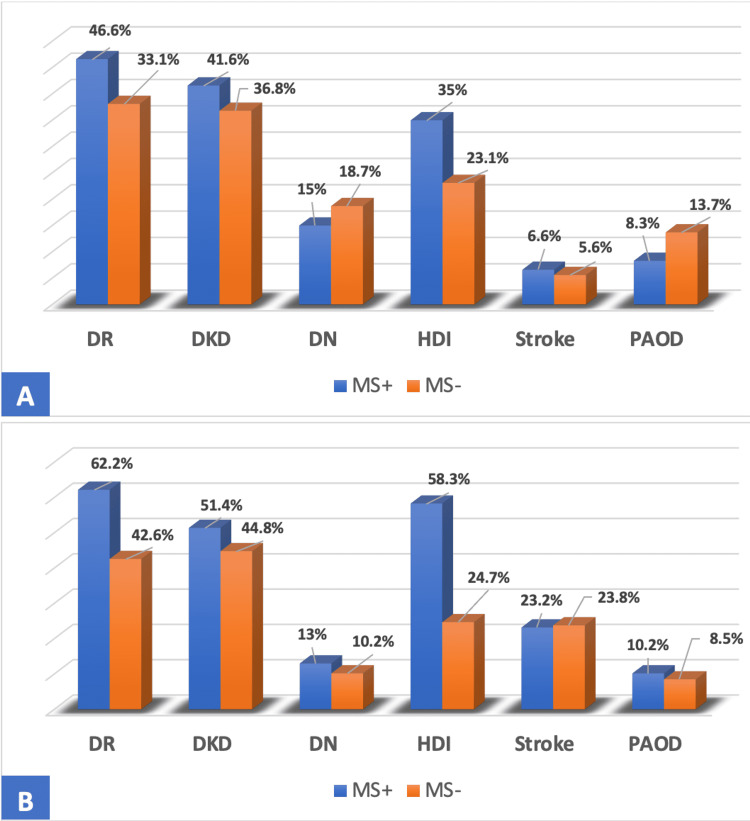
Distribution of T1D (A) and T2D (B) according to degenerative complication and MS DR: Diabetic retinopathy, DKD: Diabetic kidney disease, DN: Diabetic neuropathy, HDI: heart disease ischemic, PAOD: peripheral arterial occlusive disease

Chronic complications of diabetes in the population with MS were mainly represented by diabetic retinopathy (DR) in 62.2% of T2DMs and 46.6% of T1Ds. Concerning macrovascular complications, heart disease was the most common with a prevalence of 58.3% of T2DM with MS and 35% of T1D (Figure 1).

Frequency and combinations of MS criteria

The diagnosis of MS in our population was made due to combinations of the criteria which are summarized in Table 2. The association of the five criteria in 16.4% of our T2D patients, and 3.3% of T1D (Table 2).

**Table 2:** Number and Associative modalities of MS criteria in T2D and T1D LWC: large waist circumference. HBP: High blood pressure, HDL-C: high-density lipoprotein cholesterol, TG: triglyceridemia

The combinations of MS criteria	Type 2 diabetics	Type 1 diabetics
Association of Three criteria	44.1%	68.3 %
Diabetes + HBP + LWC	25.5%	20%
Diabetes + HDL-C + HyperTG	4.7%	18.3%
Diabetes + LWC + HDL-C	3.3%	11.6%
Diabetes + LWC + HyperTG	1.7%	6.7%
Diabetes + HBP + HyperTG	2.9%	6.7%
Diabetes + HBP + HDL-C	6%	5%
Association of Four criteria	36.6%	28.3%
Diabetes + LWC + HDL-C + hyperTG	2.1%	11.6%
Diabetes + HBP + LWC + HyperTG	12.8%	8.3%
Diabetes + HBP + LWC + HDL-C	18.2%	6.6%
Diabetes + HBP + HDL-C + HyperTG	3.5	1.6%
Association of Five criteria	19.3%	3.4%

## Discussion

The prevalence of MS differs from one study to another. The IDF and AHA/NHLBI agreed that the presence of three of the five risk factors constitutes a diagnosis of MS [7]. MS increases cardiovascular risk threefold [8]. This risk is especially increased when diabetes is present in patients with MS [9]. In Morocco, there is no large study that can provide a reliable estimate of the prevalence of MS in patients with diabetes, our study, the first in our knowledge, aimed to estimate the prevalence of MS and its different characteristics in patients with diabetes.

Our data showed that the prevalence of MS in our population was 67.5% according to the IDF 2009 definition, the same result (68.8%) was reported by Ammar et al. [10]. However, the prevalence found remains higher than that reported in Mali in 2014 with the same harmonization consensus (48.9%) [7]. Our results show a high prevalence of MS in our patients, with a remarkable predominance in T2D (78.4%), this prevalence is comparable to the results of the study carried out in Benin (79%) [11]. MS was diagnosed in 27.3% of our T1D, in this sense, a rate of 22.2% was reported in a study carried out in South India [12] and 25.5% in Germany/Austria [13]. These differences in the prevalence of MS could be explained by the difference in the study period, source population, socioeconomic differences, and the definitions used to diagnose metabolic syndrome. The prevalence of MS according to sex in our study revealed a female predominance, with a frequency of 68.7% in T2DM and 70% in T1D. These results agree with those reported by Affangla et al. in 2019 [14], this difference may be linked to menopause and lack of physical activity in women.

In our T2D, hypertension was the parameter most often observed (87.7%). It was mentioned as the most determining element in the prevalence of MS in studies conducted in Burkina Faso [15] and Ghana [16]. In T2D with MS, 44.1% had three criteria, 36.6% four criteria, and 19.3% five. These results are similar to the results of studies conducted in Guinea in 2012 [17], and Algeria [18] in 2019. In our T1D with MS, visceral obesity was the most frequent criterion (68.3%). These results contradict those reported in the Finn Diane study [19] where hypertension was the omnipresent risk factor. In T1D with MS 68.3% had three criteria, four criteria in 28.3%, and only 3.4% had five criteria for MS, our results are consistent with a study conducted in Spain [20] in 2010. The associative modality most found in our study was that of diabetes and arterial hypertension and abdominal obesity. This triad represented the most frequently observed association of MS in studies conducted on type 2 diabetics in Burkina Faso in 2016 [15], and Guinea in 2012 [17].

In our T2D patients with MS, retinopathy was the most frequent of the microvascular complications, with a frequency of 62.2% versus 42.6% of the T2D without MS (p<0.05). higher than this objectified in southern Taiwan (37.9%) [21]. These results could be explained by the presence of hypertension in 87.7% of our patients with T2D and MS. Diabetes kidney disease was present in 51.4% of cases, similar to the findings reported by Lee et al. (40.8%) [21]. Neuropathy was found in 13% of patients, this prevalence is comparable to that of studies carried out in China (16.4%) [22]. Regarding macroangiopathy, ischemic heart disease was the most frequently observed with a prevalence of 58.3% against 24.7% in patients without MS (p <0.05). This frequency is close to that shown in Brazil [23] (53%). These results support data from the literature which demonstrated that T2D who presented with MS had a cardiovascular risk, three to five times that of the non-diabetic population [24]. Stroke was found in 23.2% of cases, this rate is higher than those reported by Jing et al. [22] (10.8%), and by Lee et al. (5.8%) [21]. Arteriopathy obliterans of the lower limbs were present in 10.2%. This figure is comparable to that of the study carried out in southern Taiwan [21] with 6.1% of peripheral arterial occlusive disease (PAOD). In T1D patients with MS, microangiopathy was represented mainly by diabetic retinopathy with a prevalence of 46.6% versus 38.1% in T1D without MS (p <0.05), which may be related to the frequency of hypoHDLemia in this group (58.3%), as low levels of HDL-C have been reported as potentially contributing to the development of retinopathy [25]. This result joins that reported in the Metascreen studies [26] with 41.7% of cases. Diabetes-related kidney disease was present in 41.6% of our T1D population with MS, similar to studies in South India (48, 8%) [12], this prevalence could be explained by the frequency of abdominal obesity in our T1Ds with MS (68.3%). Neuropathy was found in 15%, similar to the results reported by Ghosh et al. (22.3%) [27]. Ischemic heart disease was the most common macrovascular complication with a rate of 35% versus 23.1% in T1D without MS (p<0.05), a prevalence of 54.2% was reported in the Metascreen [26], The prevalence of stroke was 6.6%, comparable to that reported in the study by Lee et al. (5.7%) [28]. Arteriopathy obliterans of the lower limbs was observed in 8.3% of the population; comparable to those reported by Lee et al. 14% [28].

There are currently no medicinal treatments for MS recognized by the marketing authorization. It is therefore very important to treat each of the anomalies of MS early and effectively. The aim of hygiene and dietetic measures is to modify lifestyle, fight against sedentary, increase physical activity, improve the quality of food intake (reduce excess calories), and reduce excess weight, especially abdominal weight [29]. Correcting these metabolic abnormalities reduces the underlying cardiovascular risk [30].

Limitations and strengths of the study

The sample size is less representative of the general diabetic population in the country, which makes it difficult to generalize the results. The strengths of the study are the evaluation of several parameters in a single study.

## Conclusions

Our results confirm the high prevalence of metabolic syndrome in patients with diabetes. and show that MS particularly affects women. The distribution according to the frequency of IDF criteria reveals that the most common combinations of metabolic syndrome criteria in our population were those involving diabetes combined with hypertension and high waist circumference. MS accelerates both macro and microvascular complications of diabetes. Its screening and the specific treatment of its various components are essential in order to effectively reduce the cardiovascular complications which jeopardize the functional as well as the vital prognosis of these patients.
